# Surfactant nanovesicles for augmented antibacterial activity against carbapenemase resistant enterobacteriaceae and extended spectrum beta-lactamases producing bacteria: in vitro and in vivo evaluation

**DOI:** 10.1186/s12866-023-02812-1

**Published:** 2023-03-16

**Authors:** Amal M. Abo Kamer, Noha M. Amer, Ahmed A. Abdelmegeed, Gamal M. El Maghraby, Noha M. Gamaleldin

**Affiliations:** 1 Department of Pharmaceutical Microbiology, Faculty of Pharmacy, Tanta University, Tanta, Egypt; 2 Department of Clinical Pathology, International Medical Center, Cairo, Egypt; 3 Department of Pharmaceutical Technology, Faculty of Pharmacy, Tanta University, Tanta, Egypt; 4 Department of Microbiology, Faculty of Pharmacy, The British University in Egypt (BUE), El-Sherouk, Cairo 11837 Egypt; 5 The Center for Drug Research and Development (CDRD), The British University in Egypt (BUE), El-Sherouk, Cairo Egypt

**Keywords:** ESBLs, CRE, Surfactant vesicles, *E. coli*, *K. pneumoniae*

## Abstract

The ubiquitous emergence of bacterial resistance is a challenging problem in infectious diseases treatment. Recently, new research lines employed nano-drug delivery systems to enhance antibacterial activity of the existing antibiotics. Accordingly, the objective of this study is to optimize surfactant nanovesicles to improve the antimicrobial effect of meropenem, ertapenem and tigecycline against Carbapenemase Resistant *Enterobacteriaceae* (CRE) and extended spectrum beta-lactamases producing bacteria (ESBL). *Klebsiella pneumoniae* and *Escherichia coli* were used as the test organisms. *In vivo* and *in vitro* evaluations were conducted to prove the efficacy of niosome-encapsulated drugs formulations. The results revealed that surfactant vesicles were able to reduce the MIC values of the tested drugs by nine-fold change compared to their free forms. Scanning Electron Microscope (SEM) showed possible adhesion/fusion of the vesicles encapsulated drugs on the bacterial cells compared to its solution. *In vivo* investigations using animal skin model confirmed the superiority of nanovesicles drug encapsulation regarding both wound size and histopathological examination. Wound surface area was reduced from 24.6mm^2^ in absence of drug to reach 13.9, and 6.2mm^2^ in presence of ertapenem solution or niosomes, respectively. Nanovesicular formulations can be considered as effective drug delivery systems that can diminish bacterial resistance against β-lactams antibiotics.

## Introduction

β-lactam antimicrobial agents are one of the most widely used group of antibiotics. Extensive administration of members of this group resulted in development of resistant bacterial strains. This provided special problem in case of Gram-negative bacteria worldwide. Development of resistance to this group was linked to induction of dynamic and continuous production and mutation of β-lactamases in these bacteria. This dynamic mutation broadened the spectrum of β-lactamase enzymes even to the newly developed β-lactam antibiotics. These enzymes are known as extended-spectrum β-lactamases (ESBLs) [1].

ESBLs are chemically produced by certain bacteria providing enzymatic activity against β-lactam antibiotics, rendering these drugs inactive against infections by these bacteria. Infections with ESBL-producing organisms are expected to have poor prognosis [1].

Over the past two decades, antimicrobial resistance among *Enterobacteriaceae* dramatically escalated worldwide. Bacteria belonging to the family *Enterobacteriaceae* are important pathogens in both nosocomial and community settings. *Enterobacteriaceae* are residents of intestinal flora and are the source of community acquired infections, hospital-acquired infections, sepsis, and urinary tract infections. From which bacteria such as *Escherichia coli* and *Klebsiella pneumoniae* are considered as commonly ESBLs producers [2].

The epidemiology of ESBLs is not simple as it depends on the relationship between the geographical area and the type of organism showing epidemic nature. This depends further on the behavior of the community and the health care team in hospitals. This type of resistant organism also contributes to the epidemiology of ESBLs. For example, *E. coli* is considered as endemic and *K. pneumoniae* is more epidemic [2].

The development in the class of β-lactam antibiotics introduced carbapenems, which are considered to have extended spectrum compared to traditional β-lactams with activity against Gram-negative organisms. They are very slowly hydrolyzed by most β-lactamases. Accordingly, carbapenems have been considered as proper choice in treatment of infections by ESBL bacteria. The prescription of carbapenems has been extended further to replace fluoroquinolones. This was especially evident in infections by *Enterobacteriaceae* which developed resistance to fluoroquinolones [3]. Extensive and irrational use of carbapenems resulted in the development of Carbapenem-Resistant Enterobacteriaceae (CRE). This resistance is thought to be due to secretion of carbapenemase. This is considered as a major public health threat making management of infections caused by CRE an arduous task due to the limited number of effective alternatives [4].

The frequency of development of bacterial resistance to antibiotics has been increasing in recent years. Accordingly, there is an urgent need for development of new antibiotics to overcome the developing resistance. Unfortunately, the process of new drug development is time consuming and requires high economical resources. This may add to the economic burden [5]. An alternative approach is to search for a strategy to overcome the resistance of bacteria to existing antibiotics. Development of new drug delivery systems is considered as promising tool for enhanced antibacterial activity of drugs and reduced bacterial resistance. Nano-carriers have been extensively used to deliver antibacterial agents. The goal was to enhance oral bioavailability and/or to overcome bacterial resistance [6]. Niosomes which are nano vesicular carriers showed promising results in enhanced antibacterial activity and reduced resistance against several antibiotics. These vesicular carriers comprised span with cholesterol as the main components and were able to prevent biofilm formation. These results were attributed to the ability of the nanovesicles to at least adsorb on the bacterial surface with high potential for invasion of the bacterial membrane delivering high input of drug [7]. Delivering high concentration of drug into the bacterial cells may overcome enzymatic attack but this hypothesis requires verification.

The aim of this study was to optimize niosomes as a potential drug delivery system for augmented antibacterial activity of β- lactam antibiotics against ESBLs and CRE producing bacteria.

## Materials and methods

### Materials

Ertapenem injectable powder (INVANZ^®^) manufactured by Merck Sharp & Dohme Corp., INC., Kenilworth, NJ., USA, Meropenem injectable powder (MERONEM^®^) manufactured by Pfizer Limited, NJ, UK and Tigecycline injectable powder (TYGACIL^®^) manufactured by Wyeth Lederle S.p.A, CT, Italy were purchased and used in this study. Sorbitan monostearate (Span 60) and lecithin were purchased from Sigma Chemical Co., St Louis, MO, USA. Octadecyl-amine (stearyl amine) was ordered from Sigma-Aldrich CHEMI., GmbH., Steinheim., Germany. Absolute ethanol was obtained from Fisher BioReagents, Loughborough, Leicestershire, UK. Cholesterol was purchased from AppliChem, Darmstadt, Germany. Mueller-Hinton (MH) agar and broth, tryptic Soy (TS) broth, Luria Bertani (LB) broth and MacConkey’s agar were all purchased from LAB A Neogen Company, Bury, UK. All other reactants, solvents and media were of analytical grade or higher.

### Bacterial isolates

This study was done in the Department of Microbiology, Faculty of Pharmacy at The British University in Egypt and in the Department of Microbiology, Faculty of Pharmacy, Tanta University from July 2019. Gram negative clinical isolates (110 isolates) were collected from different clinical specimens from the Clinical Pathology Department, International Medical Center, Cairo, Egypt. These specimens included pus, blood, sputum, urine, tissue cultures, wound, peritoneal fluids and vaginal swabs. *Escherichia coli* ATCC 8739 and *Klebsiella pneumoniae* ATCC 53637 were used as the quality control organisms to evaluate the MIC performance characteristics of each run.

The bacteria were stored at -20 °C and − 80 °C in LB broth supplemented with 50% glycerol. These were recovered from frozen stock by overnight culture at 37 °C on MacConkey’s agar when required.

### Identification of bacterial isolates

#### Automated VITEK 2 system

The traditional techniques for identification of bacteria and fungi include phenotyping by selection specific culture media, colony morphology, Gram staining in addition to application of alternative biochemical reactions [8]. Despite being accurate, these strategies are time consuming. Automated identification to the species level techniques have been developed with VITEK 2 being the most widely adopted technique in clinical microbiology laboratories. This technique was employed in the current study which employed VITEK 2 (BioMérieux, USA). This identification was carried out at the microbiology laboratory of Clinical Pathology Department, International Medical Center, Cairo, Egypt.

#### Capsule staining

Nigrosine dye (1 drop) was mixed with a loopful of *K. pneumoniae* bacterial suspension on glass slide before spreading as very thin film on the whole slide surface. This was achieved by dragging the liquid droplet by clean slide. The developed thin film was left to dry in the air for 5–7 min. Crystal violet was added to the slide stain the bacterial cells but not the capsules. This involved addition of the crystal violet solution for 1 min at the end of which the slide was tilted to let strain run off before leaving the slide to dry at ambient conditions. The smear was examined microscopically (100x) for the presence of encapsulated cells as indicated by clear zones surrounding the cells [9].

### Antimicrobial susceptibility testing

#### Automatic susceptibility test by VITEK 2 system

Automatic susceptibility is achieved using VITEK 2 system (BioMérieux, USA) which employs plastic reagent cards which are loaded with known amounts of antimicrobials and can accommodate microliter volumes of test media. Each VITEK test card is divided into up to 64 micro-wells, one of which is occupied by culture media only and the rest of the wells holding the predetermined quantities of the specified antibiotics combined with culture medium. Colonies from an overnight culture are dispersed in 0.45% saline to standardize the suspension to 0.5 to 0.63 McFarland. This suspension is automatically diluted and withdrawn by the system to rehydrate the antimicrobial medium within the card which is mounted in the VITEK incubator/reader. The equipment performs photometric monitoring of the growth kinetics in each well over a predetermined period (up to 18 h). Optical readings are recorded periodically (every 15 min) to quantify the amount of light passing via each well. The growth rate with reference to antibiotic concentration was employed to determine the algorithm-derived MIC which involves linear regression analysis of the growth curve. Algorithmic analysis of the growth kinetics in each well is performed by the system’s software.

#### Double disk synergy test (DDST)

Antimicrobial susceptibility of ESBLs isolates was determined according to the CLSI approved disc diffusion method [10]. Isolates from overnight culture on MacConkey agar were suspended in saline and the turbidity was adjusted to achieve a concentration equivalent to a 0.5 McFarland standard to provide a suspension containing approximately, 1 to 2 × 10 ^8^CFU/ml. A sterile cotton swab was dipped into the adjusted bacterial suspension, and firmly pressed on the inside wall of the tube above the fluid level. The swab was then used to inoculate the surface of MH agar plates by homogenous streaking over the entire sterile agar surface. The inoculated MH agar plates were left for 5 min to ensure absorption of the moisture before applying the antimicrobial disks. Each plate was loaded with 3 disks which were distributed evenly so they are no closer than 30 mm from center to center. The first disk contained amoxicillin/clavulanic acid (AMC) (20/10µg), the second contained *Cefotaxime* (30 µg) as an expanded-spectrum cephalosporin and the third contained amoxicillin (20 µg). The plates were then incubated aerobically at 37ºC for 18–24 h at the end of which the diameter of each inhibition zone was measured. The positive results were observed by a diminished susceptibility to cefotaxime was joined with a clear-cut enhancement of the inhibition zone of cefotaxime towards the AMC disk and looked as a ‘champagne-cork’ or ‘keyhole’ a distinguishing shape-zone.

#### Modified Hodge test (MHT)

Antimicrobial susceptibility of CRE isolates was determined according to the CLSI approved modified Hodge method [10]. Isolates from overnight culture on MacConkey agar were suspended in saline and the turbidity was adjusted to achieve a concentration equivalent to a 0.5 McFarland standard to provide a suspension containing approximately, 1 to 2 × 10 ^8^CFU/ml. A 0.5 McFarland dilution of the *Escherichia coli* ATCC 8739 in 5 ml of saline was prepared then diluted by 1 in 10 by saline. A sterile cotton swab was dipped into the adjusted bacterial suspension, and firmly pressed on the inside wall of the tube above the fluid level. A lawn of the 1:10 dilution of *E. coli* ATCC 8739 was streaked on a Mueller Hinton agar (MHA) plate and allowed to dry for 3–5 min. A 10 µg meropenem susceptibility disk was placed in the center of the test area. In a straight line, CRE organisms were streaked from the edge of the disk to the edge of the plate. *K. pneumoniae* ATCC 53637 was used as a QC strain. Plates were then incubated aerobically at 37ºC for 18–24 h.

#### Preparation of surfactant nanovesicles (niosomes)

Table 1 presents the composition of the prepared surfactant vesicles [7]. The vesicles were prepared by hydration of proniosmes. Span, cholesterol, lecithin and stearyl amine (if present) were melted on water bath and ethanol was added with mixing to form clear solution. Water (5ml) was added while mixing until the formation of homogenous mixture. The mixture was removed from the water bath and mixing was continued to develop proniosomes which was creamy gel. Ertapenem was solubilized in the rest of water to provide a final concentration of 3 mg/ml in the formulation. The ertapenem solution was used to hydrate the proniosomal gel to develop niosomal dispersion. This was achieved by gradual addition of ertapenem solution then water with continuous mixing. The niosomal dispersion was incubated at room temperature in the laminar flow equipment to undergo complete hydration and swelling. Swollen vesicles were then bath sonicated for 45 min to reduce the vesicle size. Heating of the samples was prevented during sonication using ice cubes. The same technique was adopted to fabricate similar vehicles [7, 11–13].

**Table 1:** The composition of niosomal formulations

Code	Composition	*Weight ratio (gm)	Size(nm)
F1	S:CH: L	4:1:1.25	129.7 (± 30.5)
F2	S:CH: L:SA	4:1:1.25:0.3	171.3 (± 11.56)

S is Span 60, CH is cholesterol, L is lecithin and SA is stearyl amine. Ethanol (5ml) was added, and the lipids were hydrated to a final volume of 100 ml with water and drug concentration was 3 mg/ml

#### Morphology and size of surfactant nanovesicles (niosomal) formulations

The morphological characteristics of niosomes were investigated by transmission electron microscope (JEM-2100, Jeol, Tokyo, Japan). Niosomal dispersion was suitably diluted with filtered water before loading one droplet onto carbon cupper plate. The sample was left to dry before staining with saturated uranyl acetate solution in 70% ethanol. The prepared sample was mounted in the holder for electron microscopical examination. Photomicrographs were acquired by proper adjustment of the magnification power. The vesicles’ size was determined by Zetasizer Nano-ZS (Malvern Instruments, UK) at 25 °C. One ml of the vesicular dispersion was diluted to 100 ml with deionized water before measurement, to have a suitable scattering intensity.

#### Determination of entrapment efficiency

The entrapment efficiency (EE%) of each drug in niosomes was determined after separation of the free drug. This was achieved by centrifugation of niosomal dispersion at 10,000 rpm at 4^o^C for 2 h. The supernatant was suitably diluted with distilled water before quantification of the free drug by UV spectrophotometry. Ertapenem, meropenem and tigecycline were quantified at 294, 298 and 245 nm, respectively. The EE% was determined by subtracting the amount of free drug from the initial amount of drug used in vesicle preparation and expressing this as percentage of the initial amount of drug added.

#### Determination of minimum inhibitory concentration (MIC)

The MIC values of free and niosomal drugs were determined using the approved Clinical and Laboratory Standards Institute [14] broth microdilution method, with slight modification [15, 16]. Ertapenem, meropenem and tigecycline stocks (3000 µg/ml) were freshly prepared in the form of aqueous drug solutions or niosomal dispersions. Each stock solution was used to prepare a series of working solutions each containing the tested drug at concentration 3 times that required in the final test concentration in the microtiter plates. This involved dilution with sterile water for the aqueous solution or with non-medicated niosomes. The final concentration was prepared by diluting the working samples 1 in 3 with MH broth. The tested final concentrations were 128, 64, 32, 16, 8, 4, 2, 1, 0.5 and 0.25 µg/ml. Aliquots (280 µl) of each concentration were loaded in the 96-well flat-bottom microtiter plates. Bacterial suspension of 0.5 McFarland turbidity standard was further diluted before adding aliquots of 20 µl to each well to achieve a final inoculum of ~ 5 × 10^5^ CFU/ml. Wells containing drug-free solutions were similarly prepared using either sterile water or drug-free niosomes in presence and absence of bacteria. This served as positive and negative controls, respectively. These microtiter plates were shaken for 5 min at the end of which the plates were incubated without shaking at 37ºC for 18 h. The MIC values were determined using resazurin which is a redox- sensitive, cell permeable, non-toxic dye which changed from blue-purple color to pink in response to bacterial growth. The technique avoids the interference of the milky niosomes [7]. Briefly, 20 µl of resazurin solution (0.1% w/v) was loaded to each well followed by 1-hour incubation at 37^o^C while shaking. Live metabolizing bacteria are expected to reduce resazurin to resorufin which is pink to red fluorescent dye. Accordingly, the lowest concentration at which the resazurin retained its original blue color was taken as a measure for the MIC [17, 18]. All experiments were conducted in triplicates to confirm the results. *Escherichia coli* ATCC 8739 and *Klebsiella pneumoniae* ATCC 53637 were used as the QC organisms to evaluate the MIC performance characteristics of each run.

#### Scanning electron microscopy (SEM)

SEM was used to monitor the morphology of bacteria before and after treatment with ertapenem solution of niosomes. This employed JEOL, JSM-5200 LV scanning electron microscope, Japan. The electron micrographs were taken after incubation of the test bacteria SEM was used to monitor the morphology of bacteria before and after treatment with ertapenem solution of niosomes. The electron micrographs were taken after incubation of the test bacterial isolates no. E35 and K41 (1 × 10^5^ CFU/ml) in Eppendorf tubes in absence or presence of ertapenem solution or niosomes. Ertapenem concentration was maintained at 0.5 MIC of each isolate. Sample preparation and procedures were conducted according to Bozzola & Russel [19]. After an overnight incubation, the bacteria were separated by 25 min centrifugation at speed of 10,000 rpm with the temperature maintained at 4^o^C. The pellets containing the bacteria were fixed in 2.5% buffered glutaraldehyde in 0.1 M PBS PH 7.4 at 4 ºC for 2 h. The fixed samples were washed three times with PBS (10 min each) before being incubated in 1% osmium tetroxide for 30 min. The pellets were then washed as before and were subjected to dehydration using increasing concentrations of ethanol (30, 50, 70, 90 ), 30 min for each concentration. The samples were dehydrated further by 4 times incubation with HPLC grade acetone. SEM, samples were dried in SPI supplies^®^, critical in point drying machine using liquid CO_2_, mounted on aluminum stubs before gold coating in a SPI Module ™ Vac/ Sputter. The coated samples were then examined using the SEM.

#### In vivo evaluation of the surfactant nanovesicles (niosomal) ertapenem antimicrobial activity

The study protocol was approved by the ethics committee for experimental, clinical, and chemical studies at Faculty of Pharmacy – The British University in Egypt with serial number EX-2001. The evaluation method was carried out in accordance with ARRIVE guidelines. A total of 33 male BALB/c mice with average weight of 25 to 30 g were employed in this study (n = 33). The mice were given free access to food and water. The mice were divided into five groups. The hair of the upper lateral part of the thigh was removed by a depilatory cream and the site was disinfected with ethanol. Bacterial suspension containing 5 × 10^8^ CFU/ml of K68 isolate in sterile Mueller Hinton agar was mixed with equal volume of sterile water (I) (n = 6), 300 µg/ml ertapenem encapsulated in niosomes F1 (II) (n = 6), 300 µg/ml ertapenem encapsulated in niosomes F2 (III) (n = 6), 300 µg/ml ertapenem solution (IV) and the last group (n = 9) divided to three subgroup A, B and C as a negative control (V). The negative control group divided to Mueller Hinton broth with equal volume of niosomes F1 (V A) (n = 3), niosomes F2 (V B) (n = 3) and sterile distilled water (V C) (n = 3). These mixtures (200 µl) were injected subcutaneously in the shaved area with groups I, II, III, IV, and V being injected with mixtures I, II, III, IV, and V, respectively. The animals were monitored for lesion development and progression and the diameter of the wounds were measured one week after development of the lesion. The animals were sacrificed 15 days after development of the lesion and samples of the lesion were taken for histopathological examination [7, 20].

#### Histopathological examination

The tissue samples were immediately preserved in 10% formaldehyde solution. The samples were subjected to dehydration using increasing concentrations of ethanol followed by washing with xylene as the terminal dehydration step. The dehydrated samples were subsequently mounted into paraffin-wax cubes before slicing into sections on glass slides. The slices were stained with hematoxylin and eosin stain (H&E) before light microscopical examination.

## Results

### Identification of bacterial isolates

**Fig. 1:**
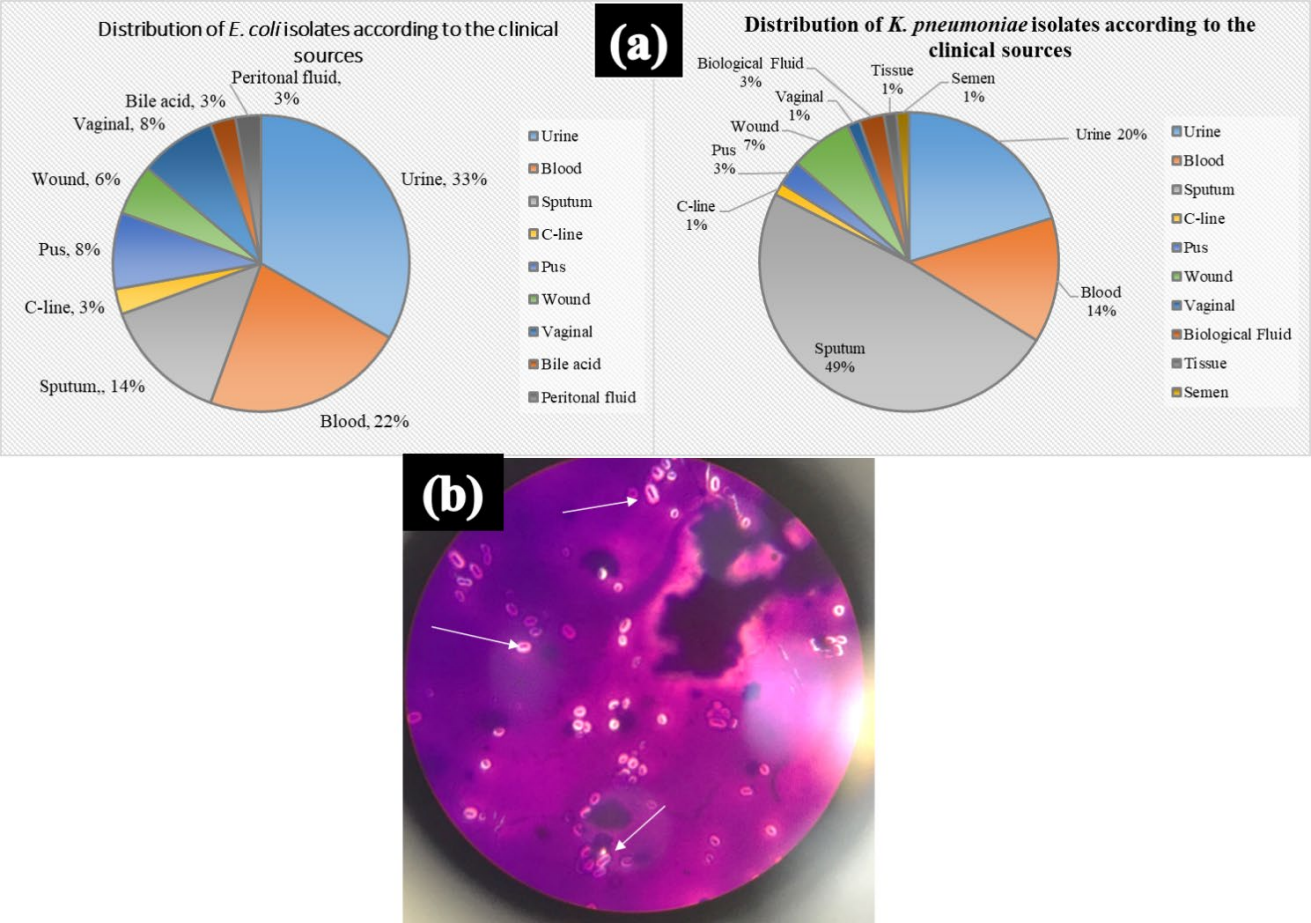
(**a**) The distribution of *E. coli* and *K. pneumoniae* isolates according to the clinical sources. (**b**) A representative photomicrograph of *K. pneumoniae* capsule. The bacterial cells appear as purple cells surrounded by clear halo. *c line: sample from the Central line. *biological fluid samples were synovial fluids

Identification of the tested isolates was made by VITEK 2 system in the clinical pathology department of the International Medical Center, Cairo, Egypt. The VITEK 2 system identification of the bacterial isolates depends on the phenotypic characterization of the biochemical reactions on the identification cards of the system. The tested isolates comprised 36 *E. coli* isolates and 74 *K. pneumoniae* isolates. The probability percent of identification of all tested isolates were ranged from 97 to 99%. The tested isolates were collected from different clinical sources including pus, blood, sputum, urine, tissue cultures, wound, peritoneal fluids and vaginal swabs. *E. coli* was shown to be prominent in urine samples (33.3%) followed by blood samples (22.2%). Whereas *K. pneumoniae* isolates were predominant in sputum samples (49%) (Fig. 1).

The isolates of *K. pneumoniae* were characterized further by light microscopy to identify the extracellular capsule which was clearly revealed after capsule staining procedures (Fig. 1).

The resistance pattern and antimicrobial susceptibility testing of the tested isolates were carried out using different techniques. These techniques include VITEK 2 automated system for determining the resistance pattern of all tested isolates and also for the detection of ESBLs producing isolates and CRE isolates. For further investigation, manual Double Disk Synergy Test (DDST) was performed for ESBLs isolates detection and Modified Hodge Test (MHT) for detection of CRE isolates.

### Automatic susceptibility test by VITEK 2 system

Susceptibility testing for all 110 isolates was performed by automated system VITEK 2. Susceptibility of the isolates measured according to the turbidity resulting from the reaction of the tested isolate with the antibiotic concentration present in each well of the susceptibility card. The amount of light transmitted through the well measured every 15 min by the system to give the optical reading. Out of the total 110 isolates 61 (55.45%) samples were ESBLs isolates. These were categorized as 33 *E. coli* organisms and 28 *K. pneumoniae* organisms. The other 49 samples (44.55%) were CRE isolates, 46 out of them were *K. pneumoniae* organisms and the other 3 isolates were *E. coli* organisms. The overall resistance pattern of all isolates against about 17 antibiotic types from separate groups was summarized for both types of microorganisms *E. coli* and *K. pneumoniae.* (Table 2). The overall resistance pattern of ESBLs isolates revealed high resistance to all beta-lactams classes antibiotics except carbapenem class. Carbapenemase producing isolates among *E. coli* and *K. pneumoniae* organisms showed great resistance to β -lactams classes and carbapenem class with high susceptibility to Tigecycline.

**Table 2:** Overall resistance pattern of the total No. of collected isolates with the antibiotics

Antibiotics	Total No. of resistant *E. coli* isolates(N = 36)	Total No. of resistant *K. pneumoniae* isolates(N = 74)
Ampicillin (AMP)	36 (100%)	74 (100%)
Ampicillin/Sulbactam (AMA)	23 (63.89)	64 (86.49%)
Piperacillin/Tazobactam (TZP)	6 (16.67%)	52 (70.27%)
Cefazolin (CZ)	36 (100%)	74 (100%)
Cefoxitin (FOX)	20 (55.56%)	51(68.92%)
Ceftazidime (CAZ)	36 (100%)	73 (98.65%)
Ceftriaxone (CRO)	36 (100%)	74 (100%)
Cefepime (FEP)	33 (91.67%)	73 (98.65%)
Meropenem (MEM)	3 (8.33%)	47 (63.51%)
Ertapenem (ERT)	3 (8.33%)	52 (70.72%)
Amikacin (AMK)	2 (5.56%)	41 (55.41%)
Gentamicin (GN)	15 (41.67%)	45 (60.81%)
Tobramycin (TOB)	12 (33.33)	50 (67.57%)
Ciprofloxacin (CIP)	28 (77.78%)	56 (75.68%)
Levofloxacin (LVX)	29 (80.56%)	56 (75.68%)
Nitrofurantoin (NIF)	3 (8.33%)	52 (70.27%)
Sulfamethoxazole/trimethoprim (SXT)	20 (55.56%)	67 (90.54%)
Tigecycline (TGI)	1 (2.78%)	10 (13.51%)

### Double disk synergy test (DDST)

**Fig. 2:**
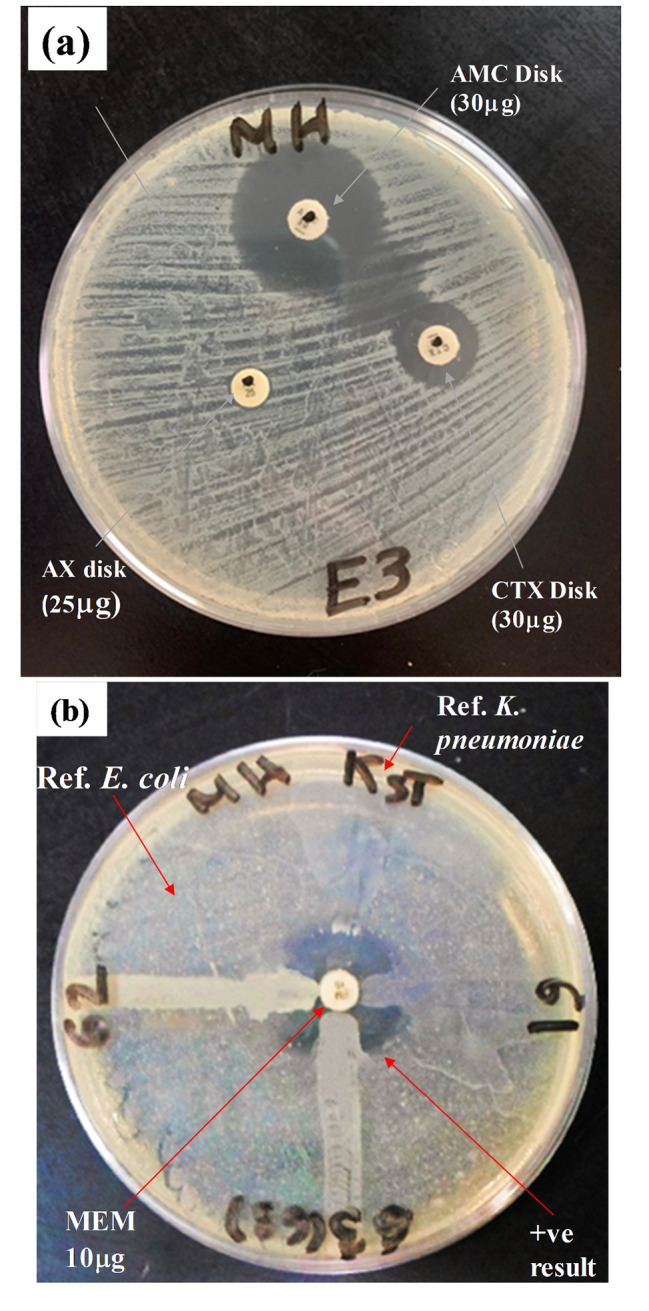
(**a**): A representative photo of positive Double Disk Synergy test for an ESBL isolate. (**b**): A representative photo of modified Hodge test contains reference strains *Escherichia coli* ATCC 8739 and *Klebsiella pneumoniae* ATCC 53637. AMC is amoxicillin/clavulanic acid 20/10 µg, CTX is cefotaxime, AX is amoxicillin and MEM is meropenem

The DDST was performed on 61 ESBLs isolates that were identified by VITEK2 to confirm their identification as ESBLs. According to the fusion pattern of the inhibition zones formed around amoxicillin/clavulanic acid disk (AMC) and the cefotaxime disk (CTX) (Figs. 2), 52 out of 61 (85.25%) isolates were confirmed as ESBLs.

### Modified hodge test (MHT)

The MHT test was applied on the 49 isolates that were identified by VITEK 2 as carbapenemase producing organisms. Forty-two out of 49 isolates (85.7%) gave positive results showing a cloverleaf like pattern within the disk diffusion zone of meropenem (10 µg) (Fig. 2). Carbapenemase producing organisms were 39 *K. pneumoniae* (93.8%) and 3 *E. coli* (6.12%).

### Characterization of surfactant nanovesicles (niosomes)

**Fig. 3:**
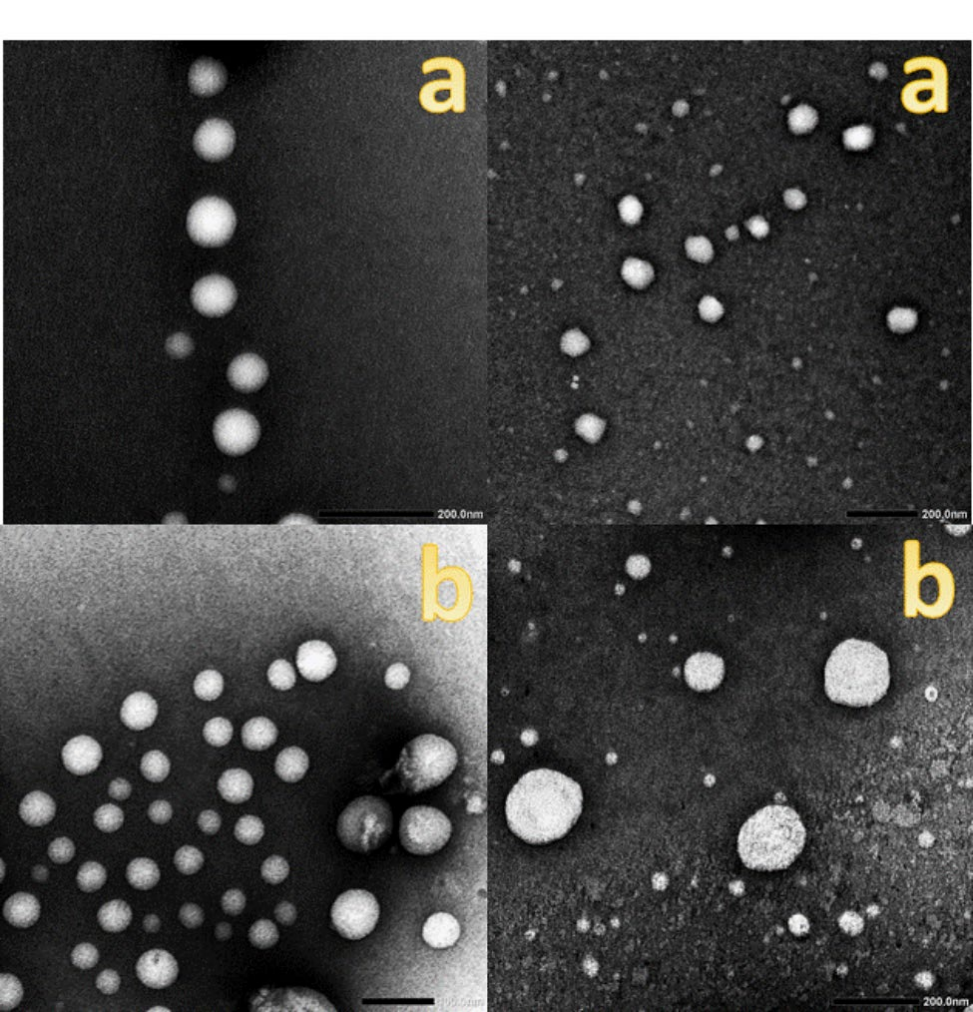
Representative photos of surfactant vesicles show their spherical appearance under Transmission Electron Microscope (TEM). (**a**) niosomal formulation 1& (**b**) niosomal formulation 2

Figure 3 shows representative transmission electron micrographs of the prepared niosomal formulations. The micrographs reflect the spherical nature of the vesicles which was revealed irrespective to the composition of the prepared formulations.

The recorded vesicle size values are in Table 1. The recorded size values were 129.7 and 171.3 nm for the plain niosomes (F1) and stearyl amine containing niosomes (F2), respectively.

The entrapment efficiency (EE%) values were 32 ± 14.2% and 36 ± 14% for ertapenem in F1 and F2, respectively. For meropenem, EE% values were 44.1 ± 13.6 and 45.9 ± 18.1% in F1 and F2, respectively. The EE% values of tigecycline were 35.6 ± 19.1 and 31 ± 8.2% in F1 and F2, respectively.

### Determination of minimum inhibitory concentration (MIC) of drugs in solution and encapsulated into surfactant nanovesicles (niosomal formulations)

**Fig. 4:**
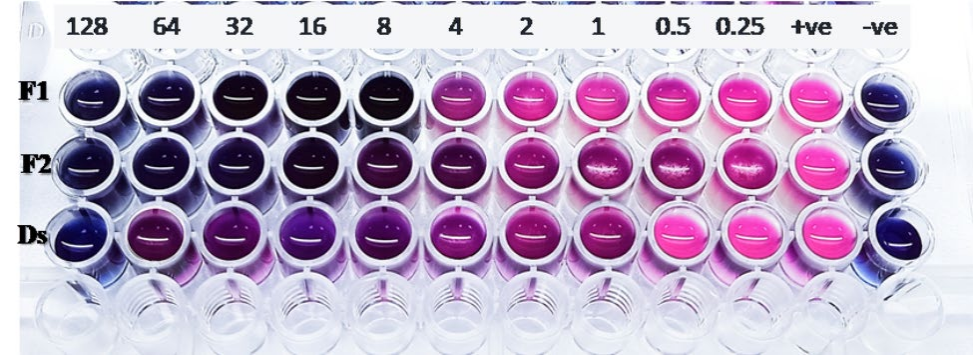
A representative photo of MIC assay. Numbers above the plate indicate the concentration of ertapenem (µg/ml) in each column, the 11th and 12th columns represent the positive and negative controls, respectively. The MICs for isolate (K68) are 8 µg/ml for ertapenem encapsulated in Formula 1 (F1), 16 µg/ml for ertapenem encapsulated in Formula 2 (F2) and 128 µg/ml for ertapenem solution (DS)

MIC determination employed resazurin dye which is a redox-sensitive indicator changing in presence of life bacteria. MIC determination was conducted based on the color change as shown in the representative plate in Fig. 4. Table 3 presents the MIC values of free and niosomes encapsulated meropenem, ertapenem and tigecycline against standard *E. coli* ATCC 8739 and *K. pneumoniae* ATCC 53637 as well as 20 selected isolates of both organisms. Selection considered the most resistant isolates. The MIC values of *E. coli* ATCC 8739 and *K. pneumoniae* ATCC 53637 were ≤ 0.25 µg/ml for the tested drugs which were prepared as aqueous solution or encapsulated in niosomes. These standard strains were taken as quality control organisms to evaluate the MIC performance in each run.

With respect to the MIC values of the tested drugs against the selected isolates of *E. coli* and *K. pneumoniae*, variable values were recorded depending on the isolates. For *E. coli*, the MIC values were in the range of > 128 to ≤ 0.25 µg/ml, > 128 to 0.5 µg/ml and 128 to ≤ 0.25 µg/ml for meropenem, ertapenem and tigecycline aqueous solutions, respectively. With respect to *K. pneumoniae* MIC values were in the range from > 128 to 32 µg/ml, > 128 to 128 µg/ml and 16 to ≤ 0.25 µg/ml for meropenem, ertapenem and tigecycline aqueous solutions, respectively.

Encapsulation of the tested drugs in niosomes reduced the MIC values of the tested antibiotics. The enhancement was noticeable in the case of resistant isolates of both *E. coli* and *K*. *pneumoniae*. Meropenem encapsulated niosomes showed one to five-fold reduction in the MIC compared to drug solution. Niosomal ertapenem reduced the MIC by one to nine-fold compared with its solution. The reduction effect of ertapenem is clear in the case of *K*. *pneumoniae* isolate No. K41 that its MIC values reduced from 128 µg/ml of solution to 2 µg/ml for nisomal formulation. For niosomes encapsulated tigecycline the reduction in MIC values were from one to seven-fold compared to its solution. The reduction in MIC was shown with *E. coli* isolate No. E35 from 128 µg/ml of solution to 4 µg/ml for nisomal formulation (Table 3). The non-medicated niosomes did not show any measurable antibacterial activity against the tested organisms irrespective to the tested isolate that appeared on the positive control wells (Fig. 4).

**Table 3:** MIC values (µg/ml) of the tested antibiotics either encapsulated in niosomal formulations or free solution

Isolate code ^(*)^:	Meropenem			Ertapenem			Tigecycline		
	**F1** ^(*)^	**F2** ^(*)^	**Solution**	**F1**	**F2**	**Solution**	**F1**	**F2**	**Solution**
E Ref. ^(*)^	≤ 0.25	≤ 0.25	≤ 0.25	≤ 0.25	≤ 0.25	≤ 0.25	≤ 0.25	≤ 0.25	≤ 0.25
E10	≤ 0.25	≤ 0.25	≤ 0.25	0.5	≤ 0.25	4	≤ 0.25	≤ 0.25	≤ 0.25
E12	≤ 0.25	≤ 0.25	≤ 0.25	≤ 0.25	≤ 0.25	1	≤ 0.25	≤ 0.25	≤ 0.25
E14	≤ 0.25	≤ 0.25	≤ 0.25	≤ 0.25	≤ 0.25	4	≤ 0.25	≤ 0.25	≤ 0.25
E16	32	32	64	≤ 0.25	≤ 0.25	1	≤ 0.25	≤ 0.25	≤ 0.25
E20	≤ 0.25	≤ 0.25	≤ 0.25	≤ 0.25	≤ 0.25	0.5	≤ 0.25	≤ 0.25	≤ 0.25
E21	≤ 0.25	≤ 0.25	≤ 0.25	≤ 0.25	≤ 0.25	0.5	≤ 0.25	≤ 0.25	≤ 0.25
E23	4	4	8	1	8	16	2	≤ 0.25	16
E35	128	128	> 128	128	> 128	> 128	4	2	128
K Ref.	≤ 0.25	≤ 0.25	≤ 0.25	≤ 0.25	≤ 0.25	≤ 0.25	≤ 0.25	≤ 0.25	≤ 0.25
K5	128	128	> 128	> 128	> 128	> 128	1	2	2
K10	32	32	64	128	128	> 128	0.5	0.5	1
K11	16	16	32	16	128	> 128	≤ 0.25	≤ 0.25	≤ 0.25
K14	128	64	> 128	> 128	> 128	> 128	≤ 0.25	≤ 0.25	≤ 0.25
K21	≤ 0.25	≤ 0.25	0.5	≤ 0.25	≤ 0.25	0.25	≤ 0.25	≤ 0.25	0.5
K22	128	128	> 128	128	> 128	> 128	≤ 0.25	≤ 0.25	≤ 0.25
K24	16	8	32	32	128	> 128	≤ 0.25	≤ 0.25	0.5
K36	32	16	64	8	64	> 128	4	2	8
K41	32	32	64	2	32	128	4	2	16
K43	128	64	> 128	> 128	> 128	> 128	≤ 0.25	≤ 0.25	≤ 0.25
K50	128	128	> 128	64	128	> 128	8	4	16
K68	128	64	> 128	8	16	128	≤ 0.25	≤ 0.25	≤ 0.25

* E Ref.: *Escherichia coli* ATCC 8729, K ref.: *Klebsiella pneumoniae* ATCC 53637, F1: niosomal formulation No. 1, F2: niosomal formulation No. 2. SD of MIC = zero in all cases

### Scanning electron microscopy (SEM)

**Fig. 5:**
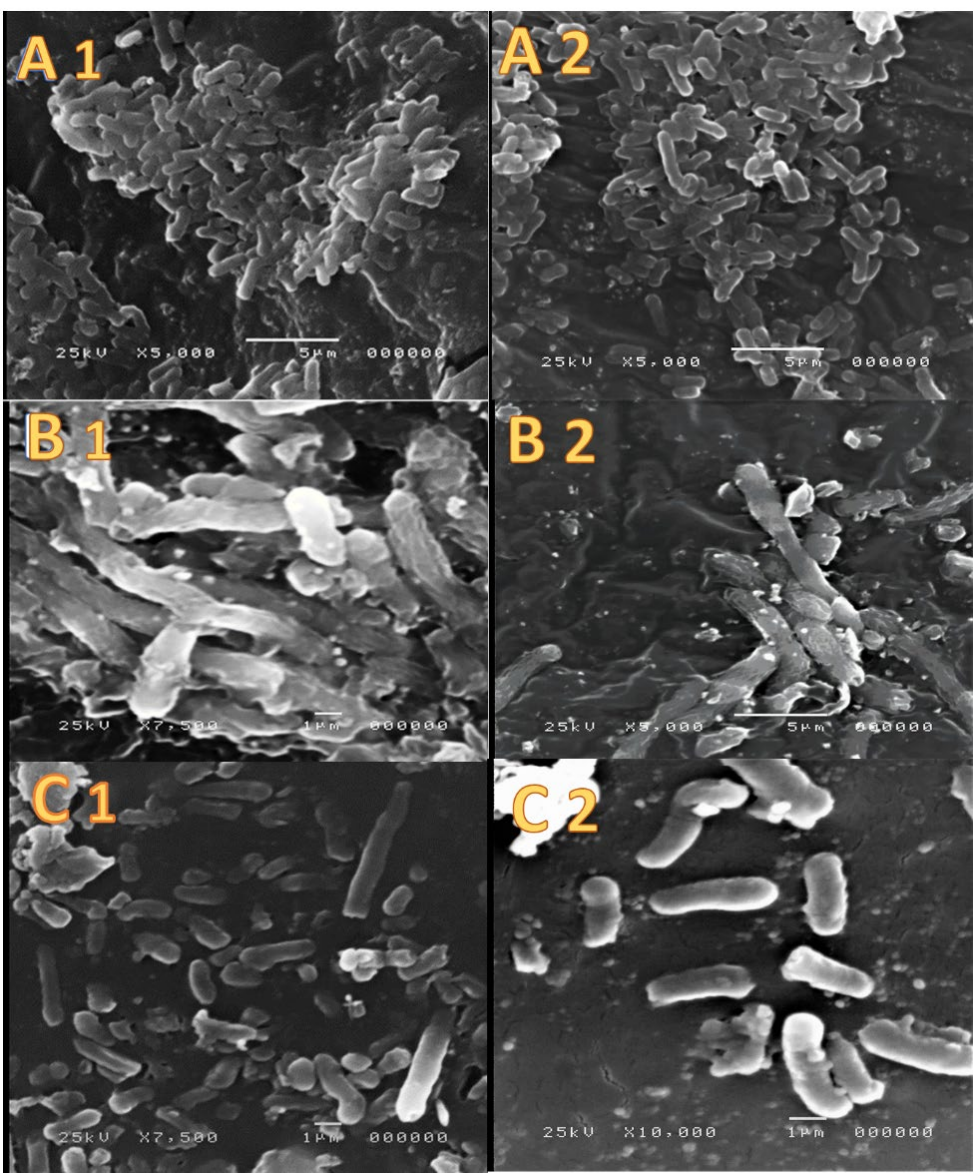
Representative scanning electron microscope graphs. 1 is for isolate No. E35 and 2 is for isolate No. K41. (A 1,2): for Normal entire bacterial cells before treatment with the ertapenem solution or encapsulated vesicles. (B 1,2). micrographs show the action of nisomal encapsulated with ertapenem on the bacterial cells resulting elongation and bursting of them with adhesion of the vesicles on their walls. (C 1,2): micrographs show the effect of the ertapenem solution on the bacterial cells that just scattered the cells from each other with elongation on some of them

Examination of the morphology of bacteria before and after treatment with ertapenem solution or encapsulated into niosomes was made microscopically by scanning electron microscope. For both *E. coli* and *K. pneumoniae*, the untreated bacterial suspensions were shown as heavy bacterial population with the bacterial cells retaining their morphological features. Treatment with drug solution reduced the density bacterial population with field showing dead cells with elongated body in addition to cells preserving their morphology. Treatment of the bacterial population with noisome encapsulated ertapenem showed unambiguous evidence for bacterial death. Interestingly, the photomicrographs showed vesicles adsorbed on the surface of bacteria (Fig. 5).

### In vivo evaluation of free and niosomal encapsulated ertapenem antimicrobial activity

**Fig. 6:**
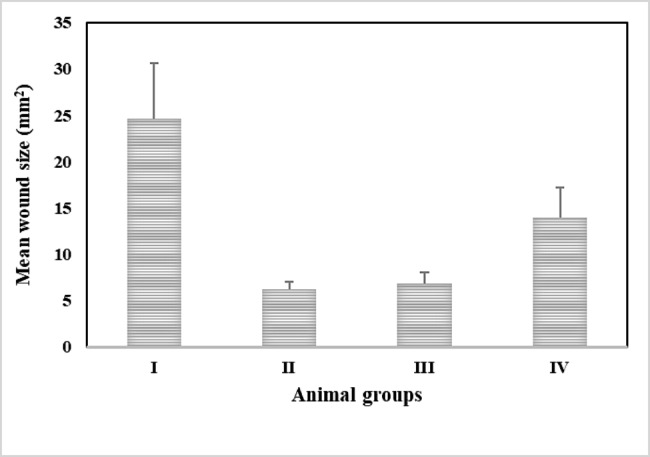
A chart of the wound size of each group with standard error, developed after induction of infection by injecting the bacterial dispersion either in water (I), ertapenem niosomes F1 (II), ertapenem niosomes F2 (III) or ertapenem solution (IV). mm^2^ stands for millimeter power of 2

**Fig. 7:**
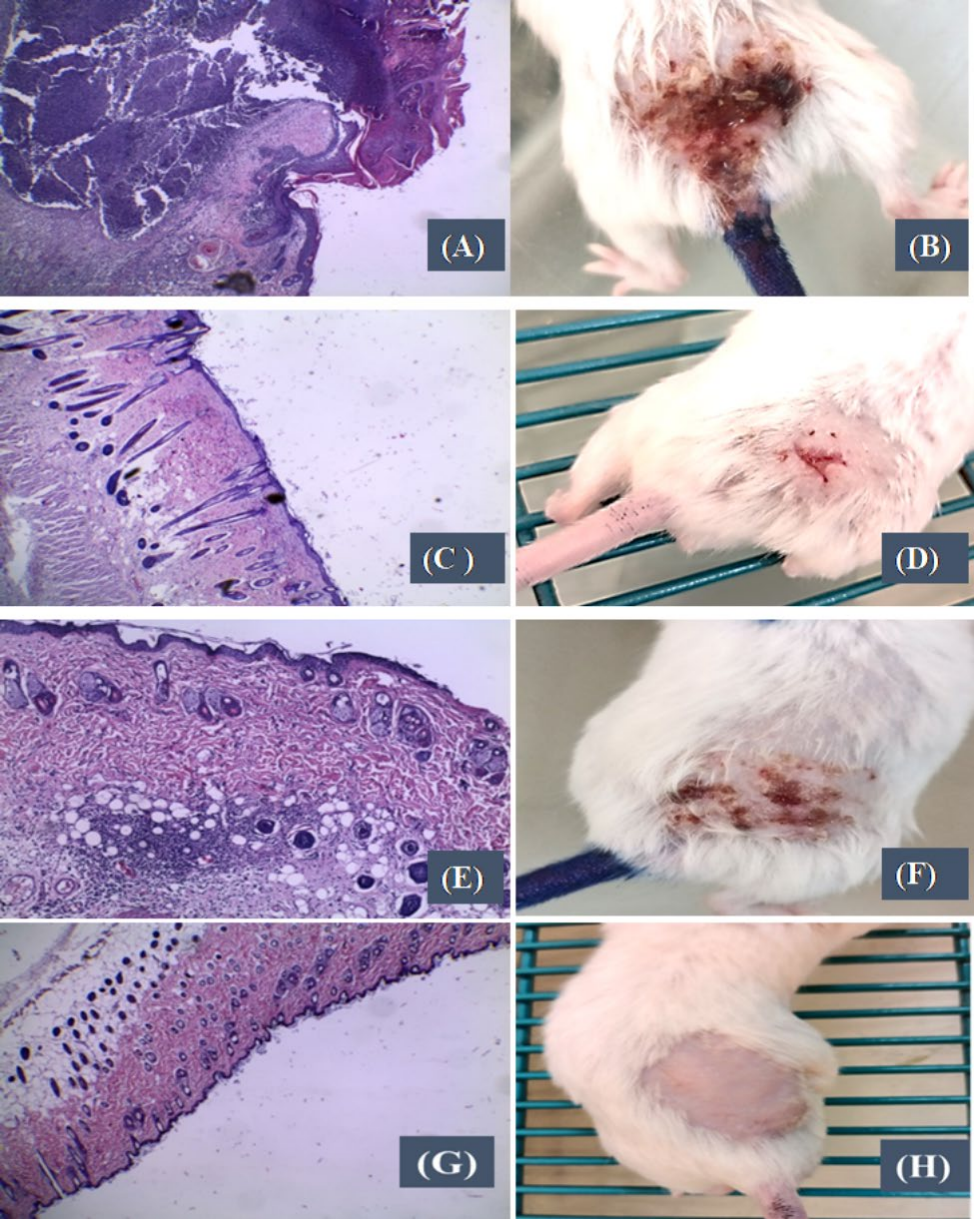
Representative photos of skin reaction of the different lesions developed after induction of bacteria as a dispersion (**A**&**B**), or with ertapenem formulations (**C**&**D**), or with ertapenem solution (**E**&**F**) and the negative intact skin (**G**&**H**) with the histopathological investigation of wound sections accordingly (hematoxylin and Eosin-stained section magnification ×120)

*In vivo* evaluation of the antimicrobial effect of ertapenem either free or encapsulate in niosomal formulations against *K. pneumoniae* (K68) isolate was carried out using BALB/c mice. The efficiency of ertapenem solution and encapsulated in niosomal formulation F1 and F2 was studied by monitoring the development of the skin wound size area on the mice. The results of the area wound size of each group were shown on the chart (Fig. 6). Histopathological examination of the skin was also conducted as additional assessment (Fig. 7). The negative control group which was injected with either sterile medium or drug free niosomal formulations did not develop any wound or any sign of inflammation at the site of injection. The histopathological examination of this group showed normal healthy skin with normal intact epidermis, normal hair follicles and the superficial dermis showed normal vascularity with no inflammatory cells (Figs. 6 and 7). For the positive control group, which was injected by bacteria in absence of ertapenem, skin wound lesions were developed seriously and appeared as inflamed reddish lesions after 5 days of induction of infection. The histopathology investigations of the produced wound in this group showed superficial epidermal ulceration with epidermal collection having acute inflammatory cells mainly polymorph nuclear leukocytes, lymphocytes and neutrophils and abscess bursting through the skin. The lesions showed thickening hyperplasia and superficial dermis with dilated blood capillaries with prominent surrounding edema (Figs. 6 and 7).

The group receiving ertapenem solution along with the bacteria showed skin wound lesions which were less inflamed reddish lesions compared with that produced in case of positive control group. The wound lesions showed superficial dermal inflammatory cell aggregates less than bacteria alone as shown in (Figs. 6 and 7).

For the groups receiving niosomal encapsulated ertapenem along with bacteria, the rate of the development of the skin wound lesions was slower as they developed after 6 days of the induction of the infection. The wound skin lesions were smaller, and the histopathology investigations showed normal intact healthy skin with normal intact epidermis, normal hair follicles and superficial dermis showed normal vascularity, no inflammatory cells. In comparison with the other skin wound lesions the niosomal formulations encapsulated ertapenem showed high reduction of development of the lesions regarding both wound size and histopathological examinations. This was evident irrespective to the composition of niosomal formulation (Figs. 6 and 7). Considering the area of the wound, statistical analysis of the data using Student T-test manifested significant reduction around the developed wound lesions in presence of ertapenem either free solution or encapsulated into niosomal formulations compared with that developed in absence of the drug (P ˂ 0.05). Niosome encapsulation significantly enhanced the efficacy of ertapenem compared with free drug solution (P < 0.05) with no significant difference being shown between the tested niosomal formulations (P > 0.05).

## Discussion

Rational use of antibiotics is essential to overcome development of resistance. Unfortunately, this is easy to say but is difficult to achieve which resulted in the development of resistance even to newly developed antibiotics. The appearance of resistant strains of the bacteria species made lots of antibiotics lose their therapeutic effectiveness especially for those patients staying in hospitals. Identification of resistant strains and development of new delivery systems which can enhance the efficacy of drugs against resistant bacteria may extend the life of existing antibacterials. In this study, most of the Extended Spectrum β-lactamases producing Enterobacteriaceae (ESBLs) and Carbapenem Resistant Enterobacteriaceae (CRE) isolates were collected from indoor hospital patients. This reflected the fact that the infections caused by ESBLs or CRE species are mutual nosocomial infections. Spreading of resistant strains can take place through exposure to hospital environment and irrational use of antimicrobial agents. Similar conclusion was reached by other investigators [21, 22].

The predominant source of *E. coli* organisms was urine samples which correlates with the fact that *E. coli* is responsible for urinary tract caused infection. As expected, most of *K. pneumoniae* isolates were recorded in sputum samples correlating with its affection to the respiratory tract. The structural features of *K. pneumoniae* with its capsule allows for easy colonization and sticking to the respiratory tract with high chance for detection in sputum samples [23].

Bacterial identification and antimicrobial susceptibility testing play an especially significant role in monitoring and reducing the bacterial resistance to antibiotics. In this study identification of bacterial isolates was carried out using automated VITEK 2 system which is reported to provide a high percentage of accuracy in identification and automated susceptibility testing with the selected isolates have probability of identifications ranged from 97 to 99%. O’Hara C. M., & Miller J. M. (2003) and Otto-Karg I et al., 2009, also reported that VITEK 2 automated system is an accurate system in identification of bacterial isolates and in antimicrobial susceptibility testing with percentage of probability 93% and 97.8% respectively [24, 25]. In this study, the collected isolates showed resistance to a variety of different antimicrobial agents using VITEK 2 system. Most of the isolates retained resistance to penicillins and cephalosporins, while only some to aminoglycoside, fluoroquinolones, and sulfa drugs. The antibiotic resistance among the collected clinical isolates reflected the growing therapeutic problem. The double disk synergy test (DDST) was performed as confirmatory test for the results which classified 61 isolates as ESBLs producing organisms by the automated VITEK 2 system. The DDST confirmed that 52 of these isolates as ESBL production. This provides 85.25% correlation which is acceptable taking into consideration the relatively shorter time of (8 h) compared to DDST, which requires 24 h to provide the results. Similar correlation was recorded by other investigators and DDST was considered as the most reliable investigation [26]. Noteworthy, 33 of the 52 confirmed ESBL producing isolates were *E. coli* and 19 organisms were *K. pneumoniae*. The predominance of ESBL production in *E. coli* compared to *K. pneumoniae* reported by other researchers [21].

Modified Hodge test (MHT) was adopted as confirmatory phenotypic method to determine carbapenemase producing isolates (CRE). MHT was recommended by the Clinical and Laboratory Standards Institute, performance standards for antimicrobial susceptibility testing as a screening method for carbapenemase [27, 28]. MHT revealed that out of 49 isolates which were classified as CRE organisms by VITEK, 42 were confirmed by the MHT method. This accounts for 85.7% success for VITEK. The recorded sensitivity of VITEK relative to MHT correlates with the published data [29]. This group concluded that the modified Hodge test was a useful method for confirming carbapenemase production.

The main objective of this study is to develop new drug delivery carrier for antibiotics with the goal of enhancing their activity against ESBLs and CRE microorganisms. This was achieved by encapsulating the drugs in niosomes which are vesicular carriers based on non-ionic surfactant with cholesterol. Niosomes have the ability to encapsulate both hydrophilic and lipophilic drugs. They have many advantages over other vesicular carriers. These include the low cost and greater physicochemical stability compared to liposomes which are the prototype of vesicular carriers [30]. Niosomes showed promising potential for enhanced antibacterial activity against biofilm forming bacteria. It was postulated that these carriers can enhance the antibiotic bacterial membrane permeability with reported adsorption on bacterial surface [7]. These findings encouraged us to select niosomes as the delivery. The current study employed niosmes made of Span 60 as the main surfactant with cholesterol and lecithin. These vesicles were prepared both in absence and presence of stearyl amine as a charging agent. Cholesterol was included as membrane stabilizer which improves drug entrapment and retention [31].

The morphology of the vesicles was spherical as expected. The recorded vesicles size values were in the nano-size range of 100–200 nm. The morphology and size values correlate with the published data [32]. The entrapment efficiency values correlate with the hydrophilic nature of the tested drugs and simulate the published work on hydrophilic drugs [32].

For the effect of niosomal encapsulation of meropenem, ertapenem and tigecycline on their antibacterial activity, the MIC was determined for the free and niosomes encapsulated drugs. The plain niosomes were used as control in this study. MIC determinations were conducted using selected resistant isolates which were characterized as ESBLs or CRE. The MIC studies employed the broth microdilution technique according to the Clinical and Laboratory Standards Institute [14] with slight modification by using redox indicator to avoid the interference from the turbidity of the milky niosomal dispersions. This technique was successfully adopted to determine the MIC for turbid nanostructure without interference [7, 33].

For drug solutions, varying degrees of resistance were recorded depending on the isolate. The recorded MIC values reflected greater magnitude in the resistance for meropenem and ertapenem in the case of *K. pneumoniae* compared with *E. coli*. This trend can be explained on the base that the *K. pneumoniae* isolates were mainly characterized as CRE bacteria while *E. coli* isolates were ESBLs except the isolates E23 and E35. The latter two isolates showed resistance for meropenem and ertapenem as reflected from the MIC values. This indicates that carbapenemase production is the main factor contributing to the resistance for meropenem and ertapenem. Tigecycline aqueous solution showed better efficacy against the CRE isolates. This result correlates with the published work which highlighted the role of carbapenemase production in the reduced efficacy of meropenem and ertapenem and recommended tigecycline as substitute for those antibiotics [34–36].

Niosomal encapsulation of drugs enhanced the antibacterial activity as reflected from reduced MIC compared with the corresponding drug solution. The magnitude of enhancement depended on the tested drug and the niosomal formulation. The recorded enhancement of the antibacterial activity after niosomal encapsulation agrees with the published data which were recorded with several types of bacteria [32, 37, 38].

The mechanism of enhanced antibacterial activity of antibiotics after encapsulation in niosomes as vesicular carriers have been researched and most reports highlighted the ability of vesicular systems to adsorb onto the bacterial cell membrane. Other studies suggested fusion of the vesicles to the bacterial surface [39, 40]. This adsorption/fusion can result in permeabilization of the bacterial membrane with subsequent increase in the drug influx into the bacterial cells. These effects are believed to minimize bacterial resistance even in biofilm forming bacteria [7, 41, 42]. The results of the current study supported the ability of niosomes to adsorb/fuse with bacterial membrane as reflected from the scanning electron microscopy. The adsorption/fusion induced increase in drug input into the bacterial cell will increase the intracellular level of drug which can subsequently increase the chance for drug to escape from the enzymatic activity. This may explain the ability of niosomes to enhance the antibacterial activity of meropenem and ertapenem against CRE bacteria.

The study was extended to monitor the effect of niosomal encapsulation of drugs on the *in vivo* efficacy of drugs. This was conducted to confirm the recorded *in vitro* enhancement in the antibacterial activity after niosomal encapsulation. Ertapenem was selected for this study employing *Klebsiella pneumoniae* isolate (K68) which was injected subcutaneously with and without treatment with drug solution or drug encapsulated niosomes. This isolate was selected for wound induction study based on the fact that it was isolated from wound and was characterized as CRE. This strategy has been successfully employed to probe the antibacterial efficacy of drug encapsulated niosomes [7]. Subcutaneous injection of the tested isolate developed skin lesion, the severity of which depended on presence or absence of the drug and the type of formulation. Induction of wound was evident after subcutaneous injection of *Klebsiella pneumoniae* to mice [20]. The recorded wound size measurement and histopathological examination confirmed the superiority of niosome encapsulated ertapenem over drug solution in reducing the ability of bacteria to induce wound. The recorded enhancement can be explained on the base of enhanced permeation of drug into the bacterial cell from niosomal dispersion. Additional explanation can be due to possible sustained effect of niosome encapsulated drug compared to the corresponding solution. The sustained effect of niosomes encapsulated rifampicin and gatifloxacin was reported by other researchers [18]. Enhanced efficacy was also reported for norfloxacin against resistant strains of *Pseudomonas aeruginosa* after niosomal encapsulation [7]. It is important to emphasize that the *in vivo* results showed good correlation with the *in vitro* data with similar effects being noticed for niosomes irrespective to the composition of the tested formulation.

## Conclusion

Niosomal formulations are promising drug carriers with a potential effect on decreasing the bacterial resistance against the antibacterial drugs. The mechanism of enhanced antibacterial activity of drug encapsulated niosomes can be due to the adhesion of vesicles on the bacterial cells’ walls with a potential for modulation of membrane permeability. The outcome of surfactant nanovesicles was evident *in vitro* and *in vivo*. However, future *in vivo* study is required in which the bacterial load in the wound is monitored.

## Data Availability

The datasets used and/or analyzed during the current study will be available from the corresponding author upon gentle request. All data and results are available upon request. All experimental protocols were approved by the faculty of Pharmacy of the British University in Egypt and Tanta University. Noha M. Gamaleldin is responsible for supplying any requested data from this study.
